# Genetic consequences of fragmentation in “*arbor vitae*,” eastern white cedar (*Thuja occidentalis* L.), toward the northern limit of its distribution range

**DOI:** 10.1002/ece3.371

**Published:** 2012-08-31

**Authors:** Huaitong Xu, Francine Tremblay, Yves Bergeron, Véronique Paul, Cungen Chen

**Affiliations:** 1 Northwest A&F UniversityShaanxi, 712100, China; 2 Chaire Industrielle CRSNG-UQAT-UQAM en Aménagement Forestier Durable, Institut de recherche sur les forêts, Université du Québec en Abitibi-TémiscamingueQuébec, J9X 5E4, Canada

**Keywords:** Boreal forest, distribution limit, genetic diversity, latitudinal gradient, microsatellite genotyping, northern edge

## Abstract

We tested the hypothesis that marginal fragmented populations of eastern white cedar (EWC) are genetically isolated due to reduced pollen and gene flow. In accordance with the central-marginal model, we predicted a decrease in population genetic diversity and an increase in differentiation along the latitudinal gradient from the boreal mixed-wood to northern coniferous forest. A total of 24 eastern white cedar populations were sampled along the north-south latitudinal gradient for microsatellite genotyping analysis. Positive *F*_is_ values and heterozygote deficiency were observed in populations from the marginal (*F*_is_ = 0.244; *P*_HW_ = 0.0042) and discontinuous zones (*F*_is_ = 0.166; *P*_HW_ = 0.0042). However, populations from the continuous zone were in HW equilibrium (*F*_is_ = −0.007; *P*_HW_ = 0.3625). There were no significant latitudinal effects on gene diversity (*H*_s_), allelic richness (AR), or population differentiation (*F*_st_). Bayesian and NJT (neighbor-joining tree) analyses demonstrated the presence of a population structure that was partly consistent with the geographic origins of the populations. The impact of population fragmentation on the genetic structure of EWC is to create a positive inbreeding coefficient, which was two to three times higher on average than that of a population from the continuous zone. This result indicated a higher occurrence of selfing within fragmented EWC populations coupled with a higher degree of gene exchange among near-neighbor relatives, thereby leading to significant inbreeding. Increased population isolation was apparently not correlated with a detectable effect on genetic diversity. Overall, the fragmented populations of EWC appear well-buffered against effects of inbreeding on genetic erosion.

## Introduction

Climate is among the most important ecological processes that strongly shape the range and genetic diversity of a species (Hewitt 2000; Thomas et al. 2004; Sexton et al. 2009; Hoban et al. 2010; Provan and Maggs 2011). The well-documented central-marginal model (Diniz-Filho et al. 2009), which is also referred to as the abundant-center model (Sagarin and Gaines 2002; Sagarin et al. 2006), predicts geographic variation in population genetic structure across a species' range (Loveless and Hamrick 1984; Yakimowski and Eckert 2008). Populations at the edge of their distribution range are subject to ecological marginality, which may affect population genetic diversity due to harsher environmental conditions (e.g., limited resources for growth and mating), isolation, and fragmentation (Diniz-Filho et al. 2009; Tollefsrud et al. 2009; Hoban et al. 2010). Fragmented populations may be prone to genetic loss and increased genetic differentiation through drift (Ellstrand and Elam 1993; Young et al. 1996; Aguilar et al. 2008). However, these responses are unlikely to be universal. Long-lived plant species, such as trees, may be buffered against genetic effects for decades or centuries (Templeton and Levin 1979; Cabin 1996; Piotti 2009). Tree species combine life-history traits that promote a high level of gene flow between populations, the maintenance of a high within-population gene diversity and low population differentiation (Hamrick et al. 1992). Thus, the genetic consequences of recent alterations to mating systems in remnant fragments are sometimes not detectable for a long time (Gamache et al. 2003).

In the boreal forests of Canada, many tree species reach their continuous distribution range at the transition between the southern mixed-wood forests, which are dominated by balsam fir (*Abies balsamea* [L.] Miller), and the northern coniferous forest, which is dominated by black spruce (*Picea mariana* [Miller] BSP). The present-day transition between these two boreal zones is controlled by both climate and fire (Bergeron et al. 2004). Mixed-wood forests are characterized by smaller and fewer severe fire events than are coniferous forests (Hély et al. 2001). Large and severe fires induce high tree mortality that results in a disadvantage to mixed-wood forest species, which generally need survivor seed trees to reinvade burned areas (Asselin et al. 2001; Bergeron et al. 2004; Albani et al. 2005).

Paleoecological records indicate that the presence of mixed-wood forest species in the coniferous forest possibly represents the remnants of formerly larger populations and would thus result from the fragmentation of those initial populations. In western Québec, post-glacial colonization occurred rapidly after the retreat of proglacial Lake Ojibway (8400 cal. BP) and involved all of the tree species that are presently found within the area (Richard 1980; Liu 1990; Carcaillet et al. 2001). Since 7000 cal. BP, balsam fir and black spruce have dominated the mixed-wood and coniferous forests, respectively (Garralla and Gajewski 1992; Gajewski et al. 1996; Carcaillet et al. 2001). The decline in the number of mixed-wood forest species could be related to the climatic shift that characterized the beginning of the Neoglacial period and the establishment of cooler and drier summers coincident with an increase in fire frequency in the coniferous forest 3000 cal. BP (Carcaillet et al. 2001; Ali et al. 2009).

**Figure 1:**
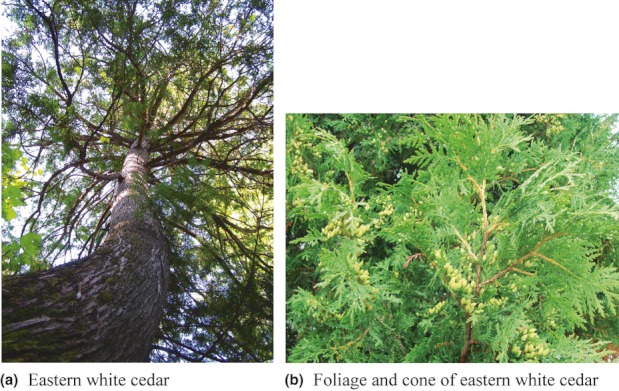
Eastern white cedar (*Thuja occidentalis* L.).

Eastern white cedar (*Thuja occidentalis* L.) (Fig. 1) is ill-adapted to fire and needs a protected area to reinvade burned areas. This species does not regenerate easily after fire, and population fragmentation following such a disturbance greatly limits its natural distribution. Eastern white cedar (EWC) reaches its northernmost distribution limit in the James Bay region of Québec at the ecotone of the mixed-wood and coniferous forest, at which point its distribution becomes increasingly sporadic as one moves northward along a latitudinal gradient.

In this study, we examined the impact of population fragmentation on the genetic diversity of EWC toward the northern edge of its range. Previous genetic studies have been based on studying allozymes and showed contrasting results. Lamy et al. (1999) reported the presence of a substantial level of genetic substructuring (*F*_st_ = 0.073) within six EWC populations. In contrast, Perry and others showed that six northern populations were not differentiated (*F*_st_ = 0.016) and, indeed, were in Hardy–Weinberg equilibrium (Perry and Knowles 1989, 1990, 1991; Perry et al. 1990).

Our main hypothesis is that populations of EWC are more genetically isolated toward the northern edge of this species' range, due to reduced pollen and gene flow between populations. We tested whether population differentiation increases and genetic diversity decreases from continuous to discontinuous and to the peripheral part of the species' distribution range. Understanding the genetic structural pattern of ecotonal populations is important because remnant marginal stands that have been eroded from larger populations that were present during the early Holocene might be at the forefront of range expansion driven by climatic changes. The amount and structure of genetic variation within these remnant populations will likely affect their potential to respond to climatic changes.

## Methods

### Study area and materials

Eastern white cedar, which is native to North America, is a wind-pollinated, monoecious, evergreen conifer species (Fowells 1965). An abundant seed crop occurs every 3–5 years, with cones opening in the autumn, but seeds may continue to fall throughout winter. Sexual maturity is generally reached at an early age, but effective seed dispersal is observed after age 20 years. Most seeds are disseminated by wind, with seed dispersal distances with estimates ranging from 45 to 60 m (Fowells 1965). Eastern white cedar is a long-lived species, which can live up to 800 years in Quebec (Archambault and Bergeron 1992).

**Figure 2:**
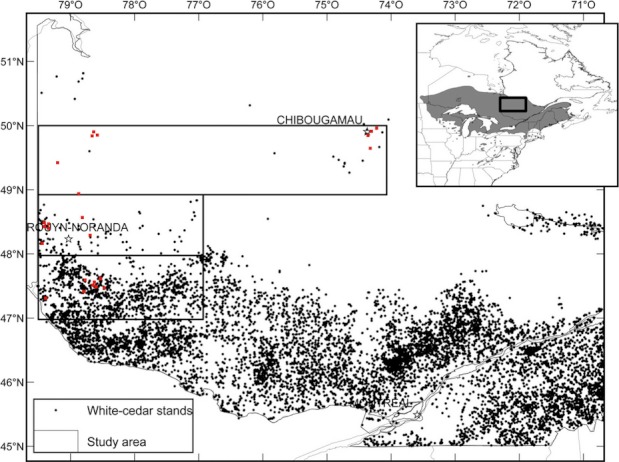
Distribution of eastern white cedar in Quebec and North America (shaded region in the map inset) and sampling sites were doted in red; the study area is divided according to bioclimatic zone (Paul 2011).

The study area is located in the Abitibi-Témiscamingue and Nord-du-Québec regions of Quebec and is divided into three bioclimatic zones based on the abundance of EWC (Fig. 2). The continuous zone falls into the balsam fir (*Abies balsamea* [L.] Mill.) and yellow birch (*Betula alleghaniensis* Britton) bioclimatic domain and represents an area where eastern white cedar is common. The discontinuous zone is in the balsam fir and white birch (*Betula papyrifera* Marsh.) bioclimatic domain and marks the northern edge of the continuous distribution, where eastern white cedar becomes less common in the forest matrix. The marginal zone is in the black spruce (*Picea mariana* [Mill.] B.S.P.) and feather moss bioclimatic domain, where only a few isolated populations are found. The site occupation rates by EWC along the gradient were estimated to 55%, 9%, and 3% in the continuous, discontinuous, and marginal zones, respectively (Paul 2011).

A total of 24 populations were selected: eight in the continuous zone (Témiscamingue, CZ1 to CZ8), seven in the discontinuous zone (Abitibi, DZ1 to DZ7), and nine in the marginal zone (Chibougamau, MZ1 to MZ4; James Bay, MZ5 to MZ9) (Table 1). Population sizes range from less than one hundred individuals in marginal and discontinuous zones to thousands of individuals in the continuous zone, with the exception of two marginal populations (MZ6, MZ2) that had 8 and 11 trees, respectively. The distance between one population and its nearest neighbor ranges from about 2 to 70 km, except for populations in Chibougamau (MZ1–MZ4), which were located about 300 km from others in the marginal zone. Between 15 and 30 trees were randomly selected in each site, for a total of about 180 trees per zone; we retained marginal populations MZ6 and MZ2 in the analysis. Foliage was collected from individual trees in each population and used for DNA analysis.

**Table 1:** Genetic variability in 24 EWC populations

Location	Pop	Latitude	Longitude	*N*	AR	*N*_a_	*N*_e_	*H*_o_	*H*_e_
Marginal zone (MZ)									
Chibougamau	MZ1	49.8754	−74.3928	21	6.1	8.0	4.6	0.655	0.756
	MZ2	49.90916	−74.3226	11	6.4	7.0	5.2	0.727	0.794
	MZ3	49.95351	−74.2291	20	6.6	8.0	6.1	0.463	0.826
	MZ4	49.64176	−74.3341	18	6.9	8.3	6.4	0.639	0.840
James Bay	MZ5	48.92772	−78.8858	30	6.6	9.5	6.2	0.661	0.815
	MZ6	49.42317	−79.211	8	5.3	5.3	4.0	0.813	0.740
	MZ7	49.85853	−78.6072	20	6.3	8.3	5.2	0.638	0.797
	MZ8	49.88349	−78.6461	25	6.7	10.0	5.1	0.680	0.798
	MZ9	49.85609	−78.6449	24	6.1	8.8	4.9	0.740	0.781
	Mean	–	–	20	6.3	8.1	5.3	0.668	0.794
	Pooled	–	–	177	11.5	11.5	7.8	0.657	0.867
Discontinuous zone (DZ)									
Abitibi	DZ1	48.5402	−78.6419	30	6.0	8.3	4.9	0.683	0.789
	DZ2	48.47015	−79.4524	24	6.1	8.3	4.8	0.625	0.789
	DZ3	48.47979	−79.4368	25	5.4	7.5	4.2	0.720	0.753
	DZ4	48.43161	−79.4018	28	5.4	8.0	4.1	0.759	0.743
	DZ5	48.26296	−78.5748	25	6.0	8.3	5.1	0.620	0.759
	DZ6	48.43101	−79.3842	25	6.4	8.5	5.3	0.630	0.805
	DZ7	48.20132	−79.4191	19	5.2	6.5	4.0	0.882	0.728
	Mean	–	–	25	5.8	7.9	4.6	0.703	0.767
	Pooled	–	–	176	11.8	11.8	6.3	0.697	0.834
Continuous zone (CZ)									
Témiscamingue	CZ1	47.42922	−78.6785	30	5.6	7.8	4.4	0.842	0.771
	CZ2	47.41669	−78.6821	27	5.2	6.8	3.9	0.796	0.712
	CZ3	47.39557	−78.7316	26	4.6	6.0	3.5	0.827	0.714
	CZ4	47.34505	−79.3926	15	4.6	5.0	3.6	0.850	0.721
	CZ5	47.3111	−78.5155	23	5.5	7.3	4.3	0.870	0.744
	CZ6	47.45395	−78.5877	30	6.2	8.8	5.6	0.883	0.801
	CZ7	47.41894	−78.6784	29	6.9	9.5	6.4	0.793	0.826
	CZ8	47.41579	−78.7117	18	6.1	8.0	5.0	0.778	0.780
	Mean	–	–	25	5.6	7.4	4.6	0.830	0.759
	Pooled	–	–	198	12.5	13.5	6.3	0.831	0.823

*N*_a_,average number of alleles per locus; *N*_e,_average number of effective alleles per locus. *H*_o_,observed heterozygosity; *H*_e_,expected heterozygosity; AR, allelic richness; *N*, number of individuals genotyped per population.

### DNA extraction, microsatellite loci amplification, and genotyping

Foliage samples were ground, and genomic DNA was extracted using the GenElute Plant Genomic DNA Miniprep Kit (Sigma-Aldrich, St. Louis, MO, USA)). Amplification was performed by a gradient polymerase chain reaction (PCR) in a total volume of 10 μL using a 96-well GeneAmp PCR System 9700 (Applied Biosystems, California, USA). Each reaction mixture contained 2.5 μL of DNA extract, 2.5 mmol/L MgCl_2_, 1 pmol each of forward and reverse primers, 0.2 μL of 10 mmol/L dNTP Mix, 1 μL 10X NovaTag Hot Start Buffer, and 0.25 U NovaTag Hot Start DNA Polymerase (Novagen PCR Kit, Madison, Wisconsin). The best results were obtained by performing a touchdown PCR that decreased the annealing temperature by 0.2°C every other cycle. At the end of each cycle, we added a final 72°C extension step. Loci developed by O'Connell and Ritland (2000) for *Thuja plicata* and by Nakao et al. (2001) for *Chamaecyparis obtusa* were utilized for microsatellite genotyping. Four loci exhibited high polymorphism (Table S1). Prior to electrophoresis, 0.5 μL of fluorescent dye-labeled PCR products were mixed with 0.25 μL of internal standard (MapMarker-1000) and 10 μL of deionized formamide. The loading products were heat denatured at 95°C for 3 min, immediately placed on ice for 5 min, and separated using capillary electrophoresis on an ABI Prism 3130 Genetic Analyzer (Applied Biosystems). Microsatellites were sized and genotyped using GeneMapper 3.7 (Applied Biosystems).

### Descriptive statistics

Micro-Checker software (Oosterhout et al. 2004) was used to detect null alleles and large allele dropouts at each locus for each population. We used the program FreeNA to estimate the frequencies of putative null alleles [r] and genetic differentiation [*F*_st_] with and without ignoring the null alleles at each locus (Chapuis and Estoup 2007). Allele frequency, allele number, and genetic estimates within populations, including the average number of alleles per locus [*N*_a_], average number of effective alleles per locus [Ne], observed heterozygosity [*H*_o_], and expected heterozygosity [*H*_e_], were calculated using GenAlex v. 6.2 (Peakall and Smouse 2006). We also calculated allelic richness [AR] using rarefaction and the inbreeding coefficient [*F*_is_] at each locus. The calculations were performed using FSTAT v. 2.9.3 (Goudet 2001). We also calculated the aforementioned genetic estimates on pooled samples for each zone. Hardy–Weinberg equilibrium was tested in each population. We also ran a global test of Hardy–Weinberg equilibrium for pooled samples from three distribution zones and for all pooled samples as a group. Bonferroni correction (Rice 1989) was applied when testing the significance of heterozygosity deficit and heterozygosity excess. All of the HW equilibrium tests were performed in FSTAT v. 2.9.3 (Goudet 2001).

### Latitudinal effects on genetic estimates

We tested for latitudinal effects by comparing differences in population genetic estimates among the three zones (marginal, discontinuous, and continuous). The genetic estimates that we compared included AR, *H*_o_ (Nei 1987), gene diversity [H_s_] (Nei 1987), *F*_is_ (Weir and Cockerham 1984), *F*_st_ (Weir and Cockerham 1984), relatedness [*R*_el_], and corrected relatedness [*R*_elc_]. We applied Hamilton's (1971) measure of relatedness, which was calculated using an estimator that was strictly equivalent to the one proposed by Queller and Goodnight (1989). To avoid bias in relatedness when inbreeding exists, we applied the corrected relatedness of Pamilo (1984, 1985). All calculations and subsequent comparisons using a permutation procedure (10,000 iterations) were performed using FSTAT v. 2.9.3 software followed the statistics of its documentation (Goudet 2001).

### Population genetic structure

To reveal genetic structure, and test if the samples could be clustered according to their respective distribution zones, we used STRUCTURE v. 2.3.2 software (Pritchard et al. 2000). Individuals were assigned to a number of assumptive clusters (assumptive groups) (K) ranging from 1 to 15 with an admixture model and the option of correlated allele frequency (Falush et al. 2003). All parameters were set following the user's manual. To choose an appropriate run length, we performed a pilot run that showed that burn-in and MCMC (Markov chain Monte Carlo) lengths of 300,000 each were sufficient to obtain consistent data. Increasing the burn-in or MCMC lengths did not improve the results significantly. Ten replicate runs for each value of *K* were carried out. The most likely value of *K* was selected by plotting ∆*K* following the ad hoc statistics (Evanno et al. 2005). The STRUCTURE results were graphically displayed using DISTRUCT (Rosenberg 2004). A neighbor-joining tree analysis (Saitou and Nei 1987) was also used to analyze the genetic structure of our samples. The neighbor-joining tree was visualized using TreeView software (D.m.page, 1996) based on Nei's standard (Nei 1987) genetic distance, Ds, calculated using the program POPULATIONS v. 1.2.30 (http://bioinformatics.org/∼tryphon/populations/). The neighbor-joining tree was bootstrapped 1000 times.

We determined the overall level of genetic differentiation using analysis of molecular variance (AMOVA) (Excoffier et al. 1992). The genetic distance matrix based on pairwise *F*_st_ (Weir and Cockerham 1984) was used to carry out the AMOVA using Arlequin v. 3.11 (Excoffier et al. 2005), with 10,000 permutations. Analysis of molecular variance was performed without grouping populations, with grouping populations by assigning them to three geographic zones, and with grouping populations by assigning them to a number of genetic groups that were identified by STRUCTURE v. 2.3.2 (Pritchard et al. 2000). We also performed a separate AMOVA on data from each of the three distribution zones. The geographic distance matrix was calculated using PASSaGE2 software (Rosenberg and Anderson 2011). A Mantel test (Mantel 1967) was applied to analyze the correlation between the geographic distance and Nei's standard genetic distance (Nei 1987). All Mantel tests were performed using GenAlex v. 6.2 (Peakall and Smouse 2006).

### Population genetic bottleneck

We tested for a recent population genetic bottleneck using the program BOTTLENECK v. 1.2.02 (Piry et al. 1999). An infinite allele model (IAM) and one-step stepwise mutation model (SMM) were applied in the bottleneck program (Cornuet and Luikart 1996). As all loci were in-between, we finally used the option of a two-phase model (TPM) (Di Rienzo et al. 1994) with 95% SMM and 5% IAM and a variance of 12, as recommended by Piry et al. (1999). Wilcoxon's test, which is better adapted to a dataset with few polymorphic loci (our case), has a robustness similar to the sign test and is as powerful as the standardized differences test, was used to test the significance of the heterozygosity excess (Piry et al. 1999). A graphical descriptor was also used to distinguish between stable and bottlenecked populations (Luikart et al. 1998). We complemented the results of heterozygosity excess and mode-shift tests with Bayesian MSVAR (Beaumont 1999; Storz and Beaumont 2002; Girod et al. 2011). MSVAR assumes that microsatellite data evolve by a stepwise mutation model and it relies on MCMC simulation to estimate the posterior distribution of parameters that describe the demographic history (Beaumont 1999). The parameters of interest in our study were current population size (*N*_0_), ancestral population size at the time population started to decline or expand (*N*_1_), and time (in generations) since population started to decline or expand (*T*). The change in population size was determined by the ratio *r* (*r* = N_0_/N_1_) where *r* < 1 indicates decline, *r* = 1 indicates stability, and *r* > 1 indicates expansion (Beaumont 1999). As the generation time for EWC is unknown, we used a value of 20 years, given that its effective seed dispersal is observed after age 20 (Fowells 1965). The exponential model was applied. The length of run for chains was determined by Raftery–Lewis statistic (Raftery and Lewis 1992, 1995). Two-hundred million iterations were sufficiently long for each chain to converge, with every 10,000th sample points being stored. The first 10% of data points were discarded from chains as burn-in to achieve stable simulations. The output was analyzed with CODA 0.14-7 package implemented in R version 2.15.0 (http://cran.r-project.org/).

## Results

### Descriptive statistics

The number of alleles per locus ranged from 10 (Locus TP10) to 17 (Locus TP12) (Table S1). Our results showed that all four loci were highly polymorphic (Table S2). The number of alleles per locus ranged from 8 at locus TP10 in the populations from the discontinuous distribution zone to 17 at locus TP12 in populations from the continuous distribution zone (Table S2). All loci exhibited positive *F*is except for locus TP10 (Table S1). MICRO-CHECKER detected the presence of null alleles at loci TP9, TP11, and TP12, and there was no evidence for large allele dropout or scoring errors due to stuttering. Null alleles occurred at very low frequencies, and similar levels of genetic differentiation (*F*st) were obtained when either excluding or not excluding the null alleles (Table S1).

At the population level, AR averaged 5.9 and ranged from 4.6 (CZ3, CZ4) to 6.9 (MZ4, CZ7). N_a_ ranged from 5.3 (MZ6) to 10.0 (MZ8), with an average of 7.8. The mean N_e_ was 4.9, with lowest value being 3.5 (CZ3) and the highest being 6.4 (MZ4, CZ7). H_o_ had a mean value of 0.7 and was lowest in population MZ3 (0.463) and highest in population CZ6 (0.883). The mean H_e_ was 0.77, ranging from 0.712 (CZ2) to 0.826 (MZ3, CZ7) (Table 1).

When populations were pooled, AR was quite similar among the three distribution zones (11.5, 11.8, and 12.5), as was Na. Ho showed an increase from the marginal zone (0.657) to the discontinuous zone (0.697), further, to the continuous zone (0.831) (Table 1). The populations from the continuous distribution zone had the highest proportion of rare alleles (frequency <1%; 0.148) and the highest total number of alleles (54) across the loci; the populations with the second highest proportion were from the discontinuous distribution zone (0.106; 47), and the populations with the least were from the marginal distribution zone (0.065; 46) (Table S2). Only populations from the continuous distribution zone had private alleles (one at locus TP10 and TP12) (Table S2).

### Latitudinal effects on genetic estimates

Among the 24 populations, seven (four marginal: MZ3, MZ4, MZ5, MZ7; three discontinuous: DZ2, DZ5, DZ6) showed a significant deficiency of heterozygotes and a departure from Hardy–Weinberg equilibrium (data not shown). None of the populations from the continuous distribution zone exhibited significant departure from HW equilibrium (data not shown). When populations were pooled, the global HW test revealed a significant departure from equilibrium and a slight heterozygote deficiency (*F*_is_ = 0.145; *P*_HW_ = 0.0125). Positive *F*_is_ values and heterozygote deficiency were also observed in populations from the marginal (*F*_is_ = 0.244; *P*_HW_ = 0.0042) and discontinuous (*F*_is_ = 0.166; *P*_HW_ = 0.0042) distribution zones. However, populations from the continuous zone were in HW equilibrium (*F*_is_ = −0.007; *P*_HW_ = 0.3625) (Table 2).

**Table 2:** Hardy–Weinberg equilibrium test

Region	*F*_is_	Heterozygosity deficit	Heterozygosity excess	*P*-value
Marginal zone	0.244	*	N/A	0.0042
Discontinuous zone	0.166	*	N/A	0.0042
Continuous zone	−0.007	N/A	ns	0.3625
Global	0.145	*	N/A	0.0125

N/A, not applicable; ns, not significant.

* *P* < 0.05.

Bonferroni corrections were applied.

The difference in H_o_ among the populations from the three zones was highly significant (*P* = 0.003), as were differences for *F*_is_ (*P* = 0.002) and *R*_elc_ (*P* = 0.005). We did not find any significant differences for AR, *H*_s_, *F*_st_, and *R*_el_ among the populations from the three zones (Table 3).

**Table 3:** Comparisons of genetic estimate differences among populations from three zones

	AR	H_o_	H_s_	*F*_is_	*F*_st_	R_el_	R_elc_
Marginal zone	6.334	0.657	0.823	0.202	0.060	0.096	−0.505
Discontinuous zone	5.805	0.697	0.786	0.112	0.070	0.119	−0.253
Continuous zone	5.589	0.831	0.777	−0.070	0.066	0.132	0.130
*P-*values	ns	**	ns	**	ns	ns	**
	(0.072)	(0.003)	(0.153)	(0.002)	(0.926)	(0.702)	(0.005)

ns, not significant.

*P < 0.05, significant.

** *P* < 0.01, highly significant.

*P-*values were obtained after 1000 permutations. AR: allelic richness. *H*_o_, observed heterozygosity; *H*_s,_ gene diversity; *F*_is_, inbreeding coefficient; *F*_st_, population differentiation; *R*_el_, relatedness; *R*_elc_, corrected relatedness.

Further comparisons revealed that the difference in H_o_ was not significant between populations from the marginal and discontinuous zones. It was significantly different between the discontinuous and continuous zones (*P* = 0.010) and between the marginal and continuous zones (*P* = 0.001) (data not shown). Similarly, the differences between populations for *F*_is_ and *R*_elc_ were only significant between the discontinuous and continuous zones (*F*_is_, *P* = 0.027; *R*_elc_, *P* = 0.052) and between the marginal and continuous zones (*F*_is_, *P* = 0.001; *R*_elc_, *P* = 0.001) (data not shown).

### Genetic structure patterning

**Figure 3:**
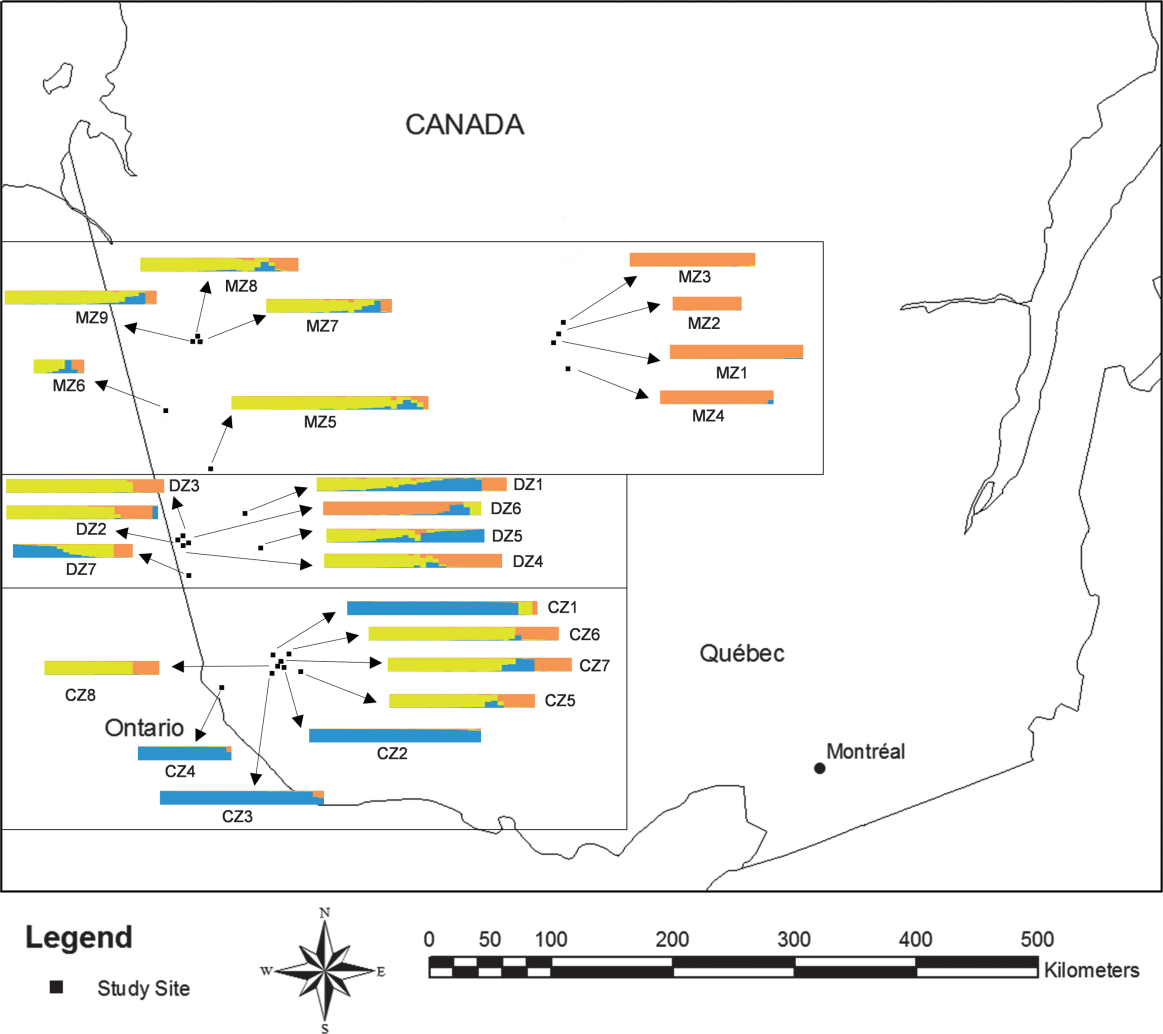
Study site, geographic origin, and genetic structure of *Thuja occidentalis* populations deduced by STRUCTURE at K = 3 (Orange cluster: MZ1, MZ2, MZ3, MZ4, and DZ6; yellow: MZ5, MZ6, MZ7, MZ8, MZ9, DZ1, DZ2, DZ3, DZ4, DZ5, CZ5, CZ6, CZ7, and CZ8; blue: CZ1, CZ2, CZ3, CZ4, and DZ7).

Bayesian analysis demonstrated the presence of population structure. The three clusters detected by STRUCTURE (Fig. S1) are displayed in orange, yellow, and blue. The largest cluster (yellow) includes 14 populations crossing the three zones (MZ5, MZ6, MZ7, MZ8, MZ9, DZ1, DZ2, DZ3, DZ4, DZ5, CZ5, CZ6, CZ7, and CZ8). The cluster depicted in blue includes five populations: four from southern sites in Témiscamingue (CZ1 to CZ4) and one from the discontinuous zone (DZ7). The cluster depicted in orange includes four populations from the northern sites (MZ1 to MZ4) and DZ6 in the discontinuous zone (Fig. 3). Most of the individuals from the marginal Chibougamau populations and population DZ6 from the discontinuous zone (Abitibi) were assigned to only one cluster. Similarly, almost all individuals from the Témiscamingue populations (CZ1 to CZ4) were assigned to only one cluster.

**Figure 4:**
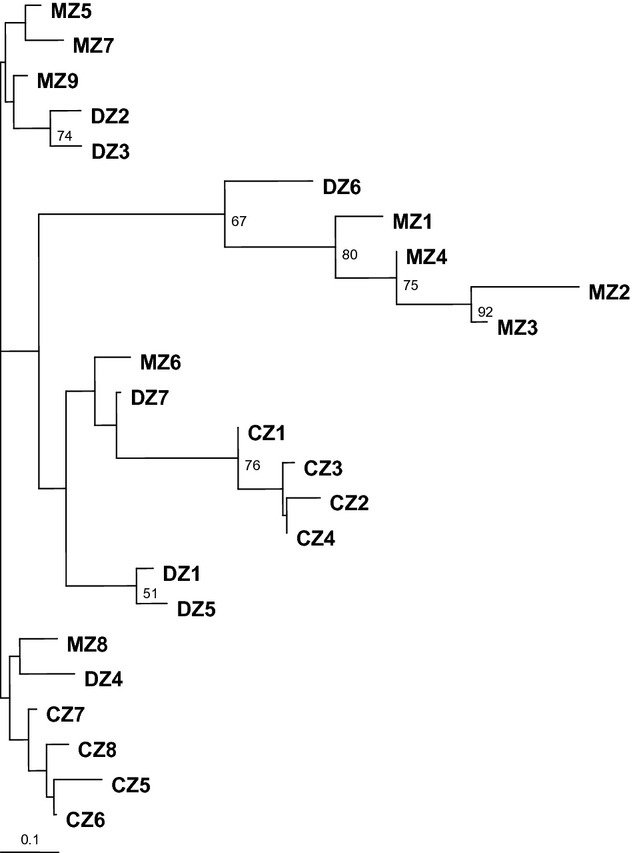
Neighbor-joining tree of *Thuja occidentalis* populations based on Nei's standard genetic distance, Ds (Nei 1987). The numbers indicate the bootstrap values; only values ≥ 50% are presented.

The results of the NJT that were based on Nei's (Nei 1987) standard genetic distance (Ds) were partially consistent with the geographic origins of the populations (Fig. 4). Four clusters can be identified at increased confidence levels (bootstrap values ≥50). Two of these clusters were also identified using STRUCTURE. MZ1, MZ2, MZ3, MZ4, and DZ6 were assigned to one cluster, while CZ1, CZ3, CZ2, and CZ4 were assigned to another cluster.

### Genetic variation partitioning

AMOVA revealed a significant level of differentiation among the EWC populations, with 7.7% of the variation found among populations and 92.3% within populations (Table 4). When the populations are pooled based on their distribution zones (marginal, discontinuous, continuous), 1.5% of the variability occurred among zones and 6.6% occurred among populations within a zone. When the populations are pooled according to the results obtained with STRUCTURE (MZ1, MZ2, MZ3, MZ4, and DZ6 (orange); MZ5, MZ6, MZ7, MZ8, MZ9, DZ1, DZ2, DZ3, DZ4, DZ5, CZ5, CZ6, CZ7, and CZ8 (yellow); CZ1, CZ2, CZ3, CZ4, and DZ7 (blue)), the variation among groups was estimated to be 7.1% and 3.4% among populations within groups. The level of variation among populations within zones was generally similar (6.0, 7.0, and 6.6%; Table 4). The variance explained by individuals within populations from the continuous zone is negative (−6.5%) and can be interpreted as being zero, which indicates an absence of genetic structure.

**Table 4:** Analysis of molecular variance for 24 populations, for populations pooled by zones (continuous, discontinuous, and marginal), for populations pooled in groups identified by STRUCTURE, and for populations at the level of each zone

Source of variation	Sum of squares	Variance components	Percentage variation	*P* value
All populations				
Among populations	176.034	0.12952	7.69904	0.00000
Within populations	904.304	0.12401	92.30096	0.00000
Pooled by zones				
Among zones	33.492	0.02610	1.51048	0.00059
Among populations within zones	142.543	0.11134	6.61151	0.00000
Within populations	904.304	0.12401	91.87801	0.00000
Groups by clusters (3) identified by STRUCTURE				
Among groups	84.830	0.12628	7.12498	0.00000
Among populations within groups	91.204	0.05736	3.39239	0.00000
Within populations	904.204	0.12401	89.48263	0.00000
Populations at the level of each zone				
Marginal				
Among populations	48.625	0.10511	6.00038	0.00000
Among individuals	334.361	0.33193	18.94962	0.00000
Discontinuous				
Among populations	46.001	0.11803	6.98706	0.00000
Among individuals	295.332	0.17632	10.43782	0.00000
Continuous				
Among populations	47.917	0.10980	6.60170	0.00000
Among individuals	274.611	−0.10815	−6.50205	1.00000

The correlation between genetic and geographic distances was positive and significant when all 24 populations were included in the analysis (Mantel test: *r* = 0.645, *P* = 0.001). However, this correlation became non-significant when the populations from Chibougamau (which are geographically distant from all other sampled populations, >300 km from the populations of James Bay) were excluded from the analysis (*r* = −0.0002, *P* = 0.571) (Fig. 3). Moreover, no significant correlation between geographic and genetic distances was detected when the IBD (isolation by distance) was tested at the level of each zone (data not shown).

### Population genetic bottleneck

A genetic bottleneck was detected by heterozygosity excess test in only one marginal population (MZ4) under both TPM and SMM models. However, population MZ4 had a normal L-shaped allelic distribution, indicating that the bottleneck was not recent or that the population is not completely isolated. Bayesian MSVAR detected a population decline in marginal population MZ8 (*r* = 0.87). Several populations (MZ3, MZ4, and MZ5) had *r*-ratios slightly below 1, which indicated a slight decline in population size (Table 5). The remaining populations showed a signal of recent expansion (*r* > 1) (Table 5).

**Table 5:** Results of MSVAR analysis of population expansion or decline

Parameter	N_0_	SE	Lower Bound	Upper Bound	N_1_	SE	Lower Bound	Upper Bound	T	S.E.	Lower Bound	Upper Bound	r-ratio
Marginal zone (MZ)													
MZ1	4.35	0.0066	3.19	5.42	4.15	0.0115	1.80	6.75	4.43	0.0148	1.29	7.91	1.05
MZ2	4.37	0.0065	3.23	5.54	4.21	0.0128	1.43	7.42	4.39	0.0150	1.45	8.07	1.04
MZ3	4.43	0.0082	2.85	6.27	4.75	0.0117	2.68	7.61	4.67	0.0180	1.08	8.67	0.93
MZ4	4.33	0.0060	3.30	5.39	4.50	0.0117	2.33	7.27	4.29	0.0154	1.32	8.24	0.96
MZ5	4.43	0.0077	3.11	6.19	4.66	0.0127	2.31	7.60	4.42	0.0175	1.21	8.61	0.95
MZ6	4.47	0.0054	3.46	5.45	3.82	0.0108	1.55	6.42	4.48	0.0120	1.74	7.25	1.17
MZ7	4.45	0.0057	3.46	5.42	3.87	0.0107	1.17	5.79	4.31	0.0133	1.71	7.72	1.15
MZ8	4.36	0.0083	2.73	6.33	5.02	0.0114	3.27	7.81	4.70	0.0191	1.12	8.91	0.87
MZ9	4.47	0.0047	3.61	5.27	3.35	0.0099	1.09	4.65	3.99	0.0115	1.57	6.79	1.33
Discontinous zone (DZ)													
DZ1	4.66	0.0030	3.98	5.30	2.41	0.0062	1.01	3.69	3.78	0.0060	2.42	5.04	1.94
DZ2	4.55	0.0043	3.79	5.33	3.25	0.0086	1.62	4.37	4.09	0.0091	2.17	5.94	1.40
DZ3	4.47	0.0053	3.47	5.45	3.56	0.0095	1.41	4.67	4.44	0.0100	2.38	6.52	1.26
DZ4	4.56	0.0033	3.85	5.25	3.29	0.0047	2.24	4.33	4.40	0.0063	2.99	5.72	1.39
DZ5	4.51	0.0032	3.81	5.20	2.62	0.0061	1.31	3.98	3.70	0.0067	2.09	5.03	1.72
DZ6	4.47	0.0029	3.82	5.07	3.05	0.0077	1.55	4.30	3.74	0.0067	2.34	5.09	1.46
DZ7	4.67	0.0030	4.01	5.35	2.57	0.0047	1.58	3.59	4.11	0.0047	3.10	5.15	1.82
Continuous zone (CZ)													
CZ1	4.48	0.0037	3.76	5.13	2.72	0.0092	0.85	4.23	3.75	0.0092	1.53	5.22	1.65
CZ2	4.18	0.0057	3.18	5.10	3.46	0.0103	0.95	4.95	4.11	0.0117	1.58	6.81	1.21
CZ3	4.28	0.0053	3.33	5.23	3.17	0.0091	0.99	4.43	4.24	0.0114	1.83	6.81	1.35
CZ4	4.30	0.0071	3.00	5.65	3.56	0.0111	0.97	5.56	4.64	0.0141	1.63	7.86	1.21
CZ5	4.28	0.0073	2.93	5.67	4.19	0.0117	1.85	6.97	4.53	0.0155	1.16	7.89	1.02
CZ6	4.54	0.0073	3.20	6.05	4.35	0.0098	2.58	6.63	4.94	0.0159	1.54	8.46	1.04
CZ7	4.72	0.0122	2.51	7.62	4.50	0.0064	3.31	5.64	4.30	0.0166	1.04	8.32	1.05
CZ8	4.74	0.0107	2.98	7.37	4.41	0.0078	3.06	6.06	4.94	0.0151	1.77	8.33	1.07

N_0_, current effective population size; N_1_, ancestral effective population size; T, time in generations since population size changes; Lower and upper bound are presented as 90% Highest Probability Density intervals.

## Discussion

Microsatellite markers revealed a significant effect of habitat fragmentation on the genetic structure in EWC populations. Populations from the marginal and discontinuous distribution ranges showed an excess of homozygotes, whereas populations from the continuous range were in HW equilibrium. Therefore, the impact of population fragmentation on the EWC genetic structure is the existence of a positive inbreeding coefficient, which was, on average, nearly 2 to 3 times higher than that of populations from the continuous zone (Table 3). This pattern could also partially reflect historical events (e.g., effects of post-glacial migration and colonization) as the farthest north population experienced population decline (Hoban et al. 2010; Dudaniec et al. 2012). This result indicated the presence of a higher occurrence of selfing within fragmented EWC populations that was coupled with a higher degree of gene exchange among near-neighbor relatives, leading to significant inbreeding. In their review, Aguilar et al. (2008) reported a trend of increased inbreeding due to habitat fragmentation; however, they reported a non-significant overall effect on *F*_is_, possibly because the fragmentation was too recent. In many published studies, the sampled adults were established before fragmentation occurred (Young et al. 1996; Lowe et al. 2005; Kettle et al. 2007). Indeed, the effect of population fragmentation on inbreeding coefficients can be detectable only after the first generation of progeny has been established.

The presence of a high level of self-fertilization in EWC has been reported in previous studies (Perry and Knowles 1990; Lamy et al. 1999). Lamy et al. (1999) showed that mating patterns are biased toward higher selfing in recently fragmented, small EWC populations. This life-history characteristic contrasts with most coniferous species, which are generally much more affected by inbreeding (Mitton 1983; Plessas and Strauss 1986; Gauthier et al. 1992; Beaulieu and Simon 1995; Ledig et al. 2000; Gamache et al. 2003; Gapare et al. 2005). A high level of inbreeding, maintained over several generations, is expected to lead to progressive genetic erosion, higher between-population differentiation and an overall decrease in genetic diversity. This pattern was not observed in this study. Genetic variation among populations was similar in the marginal, discontinuous, and continuous populations (6.0%, 7.0%, and 6.6%, respectively), as were the levels of genetic diversity (Hs, Table 3), except that only populations from continuous zones had private alleles. This is probably because the fragmentation has not progressed long enough to have detectable effects on progressive genetic erosion. Long-lived trees may be buffered against genetic erosion for centuries (Templeton and Levin 1979; Cabin 1996; Piotti 2009).

The global level of differentiation among EWC populations was relatively high and similar to that reported by Lamy et al. (1999) (7.7% vs. 7.3%) in populations sampled over a much smaller geographic area (180 km^2^). It was also higher than those values that were reported in EWC populations by Matthes-Sears et al. (1991) (1.9%) and Perry et al. (1990) (1.6%). Most alleles were distributed in populations throughout the three zones. Populations from the continuous distribution zone harbored the highest proportion of rare alleles (frequency <1%), with a decreasing trend toward the northern range margins. Yet, no significant differences were observed in allelic richness among populations from the three bioclimatic zones, indicating that populations residing in the discontinuous or marginal distribution ranges have not experienced a great decrease in population size or, if so, have overcome previous bottlenecks (Nei et al. 1975; Leberg 2002). The evidence of population decline was detected in marginal populations (MZ3, MZ4, MZ5, and MZ8). However, the detection power of our bottleneck analysis was weak due to the limited number of polymorphic microsatellite loci available for the EWC. Our results were still comparable to other studies that detected significant bottlenecks based on four polymorphic loci (Aizawa et al. 2009; Heuertz et al. 2010). Genetic bottleneck effects could also be obscured by immigration events.

The majority of studies that have examined geographic variation in genetic diversity have used a ‘categorical approach' in which only groups of peripheral and central populations were sampled (Eckert et al. 2008). Yet, the ‘categorical approach’ has also been blamed for confounding geographic position with region compared to ‘continuous sampling approach’. Our study relaxed this confounding by sampling along a latitudinal transect that encompasses central, intermediate, and peripheral populations. The geographic distribution of EWC along the latitudinal gradient was estimated from the analysis of a large inventory database (a total of 5476 sample plots) and found to decrease from 55% to 9% to 3% from the continuous to the discontinuous to the marginal zones, respectively (Paul 2011). This pattern conforms to the ‘abundant centre model’, which predicts an increase in the spatial isolation of populations from the range center toward the range limits (Sagarin and Gaines 2002; Eckert et al. 2008). This increase in population isolation was apparently not correlated with a detectable effect on genetic diversity. One plausible explanation involves the life-history characteristics of EWC. Selfing species naturally retain most of their genetic diversity within populations, and their level of population genetic diversity is less affected by restricted gene flow. Moreover, the ability of EWC to reproduce vegetatively, via layering, may buffer the genetic effects of fragmentation by delaying the time between generations (Honnay and Bossuyt 2005). A parallel study conducted at the same sites showed higher levels of layering in populations in the north (marginal and discontinuous zones) than in the south (continuous zone), with equivalent seed production along the gradient (Paul 2011). Finally, the effect of inbreeding on genetic erosion may also be buffered by selection against homozygotes in young EWC individuals, which will eliminate a higher proportion of these individuals before they become adults.

### Population structure

Both Bayesian and NJT analyses detected a certain level of genetic structure among the 24 EWC populations. Interestingly, the four marginal populations (MZ1, MZ2, MZ3, and MZ4) from Chibougamau and one population (DZ6) from Abitibi were assigned to one cluster, even though more than 400 km separated DZ6 from the Chibougamau marginal populations. One explanation may be that these populations followed the same post-glacial migration route. Apparently, the four populations from Témiscamingue (CZ1, CZ2, CZ3, and CZ4) belonged to the same cluster, indicating that gene flow (via seed or pollen dispersal) was high among them. Some sub-branches of the NJT were significant (bootstrapped values ≥50), such as the sub-branch clustering of DZ1 and DZ5 or that of DZ2 and DZ3. These populations that clustered together are genetically closer and may have followed similar post-glacial migration routes. Fourteen populations (marginal: MZ5, MZ6, MZ7, MZ8, and MZ9; discontinuous: DZ1, DZ2, DZ3, DZ4, and DZ5; and continuous: CZ5, CZ6, CZ7, and CZ8) were assigned into a single (yellow) cluster.

### Conservation implications

Our results converged to demonstrate that spatial isolation of marginal EWC populations is not associated with low genetic diversity. Therefore, increased inbreeding does not lead to a loss of genetic variation in northern EWC populations, and therefore they have the potential to respond and adapt to environmental changes. The actual distribution and expansion of white cedar at the northern edge of its range has been limited by climate in association with fires (Paul 2011). This limitation illustrates the complexity of the species' population dynamics and the difficulty of predicting future EWC distributions in a changing environment. If climate favors improved regeneration of this species and its northward migration, peripheral populations could play a major role as seed sources and in the further movement of the geographic range in response to climate changes. In contrast, if global warming triggers an increase in fire frequency (Bergeron et al. 2010), the EWC distribution could be negatively affected and reduced to lower latitudes. In such a context of uncertainty, the precautionary principle should apply, and marginal populations should be protected to allow continuity of natural evolutionary processes.

## Biosketches

Huaitong Xu is doing a Ph.D in Environmental Science at University of Quebec (UQAT-UQAM) in Canada. His research interests focus on plant population genetics including the impacts of fragmentation on tree population genetic diversity, tree regeneration patterns following forest succession, population genetic structure of tree species at their distribution limits, and landscape genetics. Dr. Yves Bergeron leads the research team in sustainable forest management, details can be found at http://web2.uqat.ca/cafd/. Author contributions: F.T., Y.B. and H.X. conceived the ideas; V.P. collected the samples; H.X. did the lab work; H.X. and F.T. analyzed the data; H.X. and F.T wrote the manuscript. C.C. gave some suggestions during the manuscript preparations.

## Supporting Information

**Table S1.** Primer characteristics, frequencies of null alleles (*r*), and *F*-statistics over all populations at each locus. *F*_st_ was estimated by harboring and excluding the null allele (INA).

**Table S2.** Allele frequencies and the number of alleles (N) by group.

**Figure S1.** Detection of the number of clusters, K, using STRUCTURE for eastern white cedar (*Thuja occidentalis* L.) populations according to Evanno et al. (2005).
