# Supplementary material for: Genetic consequences of fragmentation in “arbor vitae,” eastern white cedar (Thuja occidentalis L.), toward the northern limit of its distribution range
Source: Ecol Evol. 2012 Aug 31;2(10):2506–20. doi: 10.1002/ece3.371 (PMC3492776; doi:10.1002/ece3.371)
Supplement: Supplementary file 1 [file ece30002-2506-SD1.docx]

**Supporting Information**

**Table** **S1.** Primer characteristics, frequencies of null alleles (r), and F-statistics over all populations at each locus. F_st_ was estimated by harbouring and excluding the null allele (INA).

| Locus | Primer sequences  (5'–3') | Repeats of cloned allele | Size range  (bp) | Alleles  (n) | r | Fst | Fst  (INA) | Fis |
| --- | --- | --- | --- | --- | --- | --- | --- | --- |
|  |  |  |  |  |  |  |  |  |
|  |  |  |  |  |  |  |  |  |
| TP9 | TCTCCTTGTCTTGGATTTGG | (AC) > 20 | 123-  433 | 12 | 0.131 | 0.124 | 0.109 | 0.146 |
|  | CGGAAAGTAGTCTCATTATCAC |  |  |  |  |  |  |  |
| TP10 | TAGTTGTGTCCATTCAGGCAT | (GT)4 GC (GT)12 | 136-  156 | 10 | 0 | 0.031 | 0.038 | -0.211 |
|  | GCTCTTATCTTCTTTTAGGGC |  |  |  |  |  |  |  |
| TP11 | GCTCTTATCTTCTTTTAGGGC | (CT)12 (CA)16 | 140-  256 | 15 | 0.131 | 0.061 | 0.064 | 0.22 |
|  | CCTGATCCGCTTTGATGGGT |  |  |  |  |  |  |  |
| TP12 | GATAAGAGGCATCACTCGAG | (CA)29 | 130-  258 | 17 | 0.104 | 0.083 | 0.083 | 0.143 |
|  | CCGGATCATTAAGGGCTCTA |  |  |  |  |  |  |  |
| All loci | - | - | - | - | - | 0.0756 | 0.0724 | - |

**Table** **S2.** Allele frequencies and the number of alleles (N) by group.

| Locus | Allele | Marginal | Discontinuous | Continuous |
| --- | --- | --- | --- | --- |
| TP9 | N | 9 | 11 | 12 |
|  | 123 | - | 0.023 | 0.010 |
|  | 139 | 0.081 | 0.099 | 0.066 |
|  | 141 | - | 0.060 | 0.020 |
|  | 143 | 0.073 | 0.054 | 0.038 |
|  | 147 | - | 0.020 | 0.003 |
|  | 159 | 0.048 | 0.014 | 0.010 |
|  | 211 | 0.132 | 0.045 | 0.010 |
|  | 215 | 0.126 | - | 0.013 |
|  | 257 | 0.135 | 0.210 | 0.250 |
|  | 259 | 0.039 | 0.014 | 0.073 |
|  | 431 | 0.174 | 0.213 | 0.303 |
|  | 433 | 0.191 | 0.247 | 0.205 |
| TP10 | N | 9 | 8 | 10 |
|  | 136 | 0.051 | 0.043 | 0.053 |
|  | 138 | 0.056 | 0.031 | 0.033 |
|  | 140 | 0.056 | - | 0.008 |
|  | 142 | 0.062 | 0.023 | 0.003 |
|  | 144 | 0.149 | 0.205 | 0.285 |
|  | 146 | 0.202 | 0.284 | 0.152 |
|  | 148 | 0.292 | 0.301 | 0.348 |
|  | 150 | 0.126 | 0.085 | 0.101 |
|  | 152 | 0.006 | 0.028 | 0.010 |
|  | 156 | - | - | 0.008 |
| TP11 | N | 14 | 13 | 15 |
|  | 140 | 0.098 | 0.188 | 0.028 |
|  | 142 | 0.025 | 0.017 | 0.013 |
|  | 158 | 0.045 | 0.040 | 0.008 |
|  | 160 | 0.062 | - | 0.005 |
|  | 166 | 0.157 | 0.122 | 0.258 |
|  | 180 | 0.059 | 0.034 | 0.109 |
|  | 182 | 0.034 | 0.006 | 0.071 |
|  | 200 | 0.065 | 0.009 | 0.043 |
|  | 202 | 0.053 | 0.048 | 0.040 |
|  | 212 | 0.090 | 0.139 | 0.093 |
|  | 214 | 0.045 | 0.031 | 0.015 |
|  | 220 | - | 0.003 | 0.035 |
|  | 222 | 0.183 | 0.233 | 0.255 |
|  | 224 | 0.039 | - | 0.008 |
|  | 256 | 0.045 | 0.131 | 0.020 |
| TP12 | N | 14 | 15 | 17 |
|  | 130 | 0.045 | 0.043 | 0.015 |
|  | 138 | 0.081 | 0.023 | 0.030 |
|  | 140 | 0.143 | 0.119 | 0.086 |
|  | 142 | 0.216 | 0.250 | 0.096 |
|  | 144 | 0.174 | 0.102 | 0.061 |
|  | 146 | 0.025 | 0.006 | 0.028 |
|  | 148 | 0.062 | 0.057 | 0.063 |
|  | 150 | 0.056 | 0.037 | 0.030 |
|  | 156 | 0.039 | 0.102 | 0.061 |
|  | 158 | 0.062 | 0.020 | 0.018 |
|  | 160 | - | 0.009 | 0.015 |
|  | 202 | 0.037 | 0.014 | 0.018 |
|  | 214 | 0.048 | 0.102 | 0.005 |
|  | 240 | - | 0.037 | 0.146 |
|  | 242 | - | - | 0.058 |
|  | 256 | 0.006 | 0.080 | 0.184 |
|  | 258 | 0.006 | - | 0.086 |
| N (total allele) | | 46 | 47 | 54 |
| The Rate of Rare Alleles (Frequency < 1%) | | 0.065 | 0.106 | 0.148 |

**Fig. S1.** Detection of the number of clusters, K, using STRUCTURE for eastern white cedar (*Thuja occidentalis* L.) populations according to Evanno et al. (2005).
